# Bladder cancer patients have increased NETosis and impaired DNaseI-mediated NET degradation that can be therapeutically restored *in vitro*

**DOI:** 10.3389/fimmu.2023.1171065

**Published:** 2023-05-19

**Authors:** Raquel Herranz, Julia Oto, Marta Hueso, Emma Plana, Fernando Cana, María Castaño, Lourdes Cordón, David Ramos-Soler, Santiago Bonanad, César D. Vera-Donoso, Manuel Martínez-Sarmiento, Pilar Medina

**Affiliations:** ^1^ Haemostasis, Thrombosis, Arteriosclerosis and Vascular Biology Research Group, Medical Research Institute Hospital La Fe, Valencia, Spain; ^2^ Angiology and Vascular Surgery Service, La Fe University and Polytechnic Hospital, Valencia, Spain; ^3^ Hematology Research Group, Medical Research Institute Hospital La Fe, CIBERONC (CB16/12/00284), Valencia, Spain; ^4^ Department of Pathology, La Fe University and Polytechnic Hospital, Valencia, Spain; ^5^ Thrombosis and Haemostasis Unit, Haematology Service, La Fe University and Polytechnic Hospital, Valencia, Spain; ^6^ Urology Service, La Fe University and Polytechnic Hospital, Valencia, Spain

**Keywords:** biomarkers, bladder cancer, DNaseI, Dornase alfa, FFPE tissue, NETs, neutrophil extracellular traps, Pulmozyme^®^

## Abstract

**Background:**

Neutrophils, key players of the immune system, also promote tumor development through the formation of neutrophil extracellular traps (NETs) in a process called NETosis. NETs are extracellular networks of DNA, histones and cytoplasmic and granular proteins (calprotectin, myeloperoxidase, elastase, etc.) released by neutrophils upon activation. NETs regulate tumor growth while promoting angiogenesis and invasiveness, and tumor cells also stimulate NETosis. Although NETosis seems to be increased in cancer patients, an increase of NETs in plasma may also be mediated by an impaired degradation by plasma DNaseI, as evidenced in several immunological disorders like lupus nephritis. However, this has never been evidenced in bladder cancer (BC) patients. Herein, we aimed to evaluate the occurrence of increased NETosis in plasma and tumor tissue of BC patients, to ascertain whether it is mediated by a reduced DNaseI activity and degradation, and to *in vitro* explore novel therapeutic interventions.

**Methods:**

We recruited 71 BC patients from whom we obtained a plasma sample before surgery and a formalin-fixed paraffin embedded tumor tissue sample, and 64 age- and sex-matched healthy controls from whom we obtained a plasma sample. We measured NETs markers (cell-free fDNA, calprotectin, nucleosomes and neutrophil elastase) and the DNaseI activity in plasma with specific assays. We also measured NETs markers in BC tissue by immunofluorescence. Finally, we evaluated the ability of BC and control plasma to degrade *in vitro*-generated NETs, and evaluated the performance of the approved recombinant human DNaseI (rhDNaseI, Dornase alfa, Pulmozyme^®^, Roche) to restore the NET-degradation ability of plasma. *In vitro* experiments were performed in triplicate. Statistical analysis was conducted with Graphpad (v.8.0.1).

**Results:**

NETosis occurs in BC tissue, more profusely in the muscle-invasive subtype (*P*<0.01), that with the worst prognosis. Compared to controls, BC patients had increased NETosis and a reduced DNaseI activity in plasma (*P*<0.0001), which leads to an impairment to degrade NETs (*P*<0.0001). Remarkably, this can be therapeutically restored with rhDNaseI to the level of healthy controls.

**Conclusion:**

To the best of our knowledge, this is the first report demonstrating that BC patients have an increased NETosis systemically and in the tumor microenvironment, in part caused by an impaired DNaseI-mediated NET degradation. Remarkably, this defect can be therapeutically restored *in vitro* with the approved Dornase alfa, thus Pulmozyme^®^ could become a potential therapeutic tool to locally reduce BC progression.

## 1 Introduction

Bladder cancer (BC) is the 12^th^ most frequent cancer worldwide, with an incidence of more than half a million new cases per year, in 2020. It ranks 14^th^ regarding mortality from cancer per year, provoking more than 200,000 deaths in 2020. This corresponds to an increase of 4% in incidence and a 6% in mortality compared to 2018 (1).

Neutrophils are the most abundant circulating leukocytes in humans (50-70%). Increased neutrophil counts have been associated with poor prognosis in several cancer types including BC (2–4). In fact, a high neutrophil infiltrate has been observed in the tumor microenvironment (TME) of BC, among other cancer types, which is associated with unfavorable outcomes (5–7).Neutrophils are the first responders in the innate immune system, as these are in charge of clearing pathogens by phagocytosis, degranulation and by neutrophil extracellular trap (NET) formation, upon activation. NETs are extracellular networks composed of DNA, histones and granule and cytoplasmic proteins (calprotectin, myeloperoxidase or elastase, among others) released by neutrophils in a process called NETosis (8, 9). NETs trap pathogens at the site of injury, but also trigger coagulation. Moreover, NETs inhibit natural anticoagulants (like protein C) further prompting thrombosis. In cancer-associated thrombosis, cancer cells induce an increase in peripheral blood neutrophils prone to NETosis and also activate neutrophils to produce more NETs than those activated by other means (10). Consequently, an increase in NETs has been evidenced in patients with thrombosis and cancer-associated thrombosis (11–14). Regarding tumorigenesis, neutrophils are actively attracted by chemokines to the TME, the so-called tumor-associated neutrophils (TANs), where NETs are prompted (15). In the TME, neutrophils display plasticity, either promoting or inhibiting tumor growth depending on cytokine signaling, epigenetic modifications and other factors present in the TME that can modify the function and morphology of neutrophils. On the one hand, NETs enhance tumor growth, angiogenesis and invasiveness by favoring the extravasation of tumor cells to the bloodstream, covering them with platelets to help them escape the immune response, escorting circulating tumor cells to the metastatic niche and even awakening dormant cancer cells to promote tumor growth (16, 17). In fact, the presence of NETs within tumors has been related to poor outcomes of cancer patients (18, 19). On the other hand, NETs also reduce the threshold of T cell activation thereby promoting an adaptive immune response that may help eliminate cancer cells and exert direct cytotoxicity on tumor cells by releasing reactive oxygen and nitrogen species, among other molecules (16). These opposed roles of neutrophils/NETs in cancer may be explained by the recent phenotypical sub-classification of neutrophils in N1 and N2. N1 neutrophils display anti-tumor activity by releasing pro-inflammatory or immune-stimulatory cytokines and N2 display pro-tumor properties due to immunosuppressive and tumor-promoting activity (20). This neutrophil plasticity is regulated by the different molecules present in the TME, which determines the resistance to oncologic treatment (15, 21).

To date, scarce studies have explored the role of NETs in BC, and these focus on the role of NETs in treatment outcome (22, 23). Although the impact of NETs has been investigated in multiple aspects of tumorigenesis, an increased NETosis has never been evidenced in plasma or in the tumor microenvironment of BC patients. Ours is the first study that demonstrates an increase of NETs in plasma of BC patients, and we have elucidated that this effect is in part due to an impaired NET degradation caused by a deficiency in plasma DNaseI. More importantly, we demonstrate for the first time that this deficiency can be therapeutically restored *in vitro*. Furthermore, we demonstrate the occurrence of NETosis in the tumor microenvironment, which is exacerbated in muscle-invasive BC (MIBC) patients compared to non-muscle invasive BC (NMIBC).

## 2 Materials and methods

### 2.1 Study subjects

A total of 71 patients of different BC stages and 64 age- and sex-matched healthy controls, who underwent an ultrasound scan to rule out the presence of urological malignancies or other alterations, were recruited between July 2016 and March 2022 at La Fe University and Polytechnic Hospital (Valencia, Spain) and were followed until July 2022.

Pre-operative clinical staging was performed through physical examination, urine cytology and CT scans of the chest, abdomen and pelvis (in case of invasive BC). The tumor histological classification was done according to grade in the WHO 1973 classifications. Demographic and clinical data were collected.

The exclusion criteria were lack of informed consent, absence of histological confirmation and presence of other malignancies.

All participants provided written informed consent according to protocols approved by the ethics review board at La Fe University and Polytechnic Hospital (ref. 2014/0314). The study was performed according to the declaration of Helsinki, as amended in Edinburgh in 2000.

### 2.2 Sample collection

Blood was drawn from all patients before surgery. Blood was collected in 4.5 mL Vacutainer tubes (BD Diagnostics, Franklin Lakes, NJ, USA) containing 0.5 mL of 0.109 M trisodium citrate. Platelet-free plasma was obtained by centrifugation at 1.811 x g for 30 min at 4°C and stored in aliquots at -80°C until further use.

For neutrophil isolation, 18 mL blood were collected from a healthy control in tubes containing K3EDTA on the day of the isolation.

Formalin-fixed paraffin-embedded (FFPE) BC tissue sections were obtained from 30 patients.

### 2.3 Quantification of NETs and inflammatory markers in plasma

Different markers of NETs were quantified in the plasma sample obtained before surgery, following the strategy addressed in previous studies (11–13), according to manufacturer´s instructions. Namely, cell-free DNA (cfDNA, Quant-iT PicoGreen dsDNA kit, Life Technologies, Eugene, OR, USA), nucleosomes (Cell Death Detection ELISA^PLUS^ kit, Roche, Mannheim, Germany), calprotectin (Human Calprotectin ELISA kit, Hycult Biotech, Uden, The Netherlands), myeloperoxidase (MPO, Human MPO ELISA kit, Abnova, Taoyuan, Taiwan) and neutrophil elastase (NE, PMN Elastase Human ELISA Kit, Abcam, Cambridge, UK).

As inflammation marker, we quantified interleukin 6 (IL-6) (Human IL-6 High Sensitivity ELISA Kit, Diaclone, Besançon, France) following manufacturer´s instructions.

### 2.4 Quantification of DNaseI activity in plasma

DNaseI activity was measured by the single radial enzyme diffusion (SRED) assay as follows: a solution of 20 mM Tris-HCl pH 7.8 containing 10 mM MgCl_2_, 2 mM CaCl_2_ and 45 mg/mL DNA from salmon testes (Sigma-Aldrich, Saint Louis, MO, USA) was prepared. The DNA solution was heated at 50°C for 10 min and mixed with an equal volume of 2% ultra-pure agarose (Condalab, Torrejón de Ardoz, Madrid, Spain) and 16 µL Safeview (NBS Biologicals, Huntingdon, UK). Forty mL of the mixture were poured into a Petri dish and let solidify before making 1-mm diameter wells. Two µL plasma were incubated into each well for 17 h at 37°C in a humid chamber. Samples were tested in duplicates. The fluorescence of the gels was recorded in a transilluminator (Uvitec, Cambridge, UK) and the area of the degradation halo was measured with ImageJ (NIH, https://ij.imjoy.io/). DNaseI activity of each plasma sample was calculated by interpolating with a standard curve prepared with known increasing DNaseI activity units.

### 2.5 Neutrophil isolation

Three mL Lymphoprep (STEMCELL Technologies, Saint Égrève, France) were layered onto 3 mL Histopaque 1119 (Sigma-Aldrich) and 8 mL blood anticoagulated with EDTA were carefully layered on top. After centrifugation at 700 × g for 30 min with minimum speed for acceleration and break, the polymorphonuclear cells were recovered at the interface between Histopaque-1119 and Lymphoprep, transferred to a new tube and washed with 1X PBS (Gibco, Life Technologies). Contaminant erythrocytes were lysed with 1 mL Cell Lysis Solution (Promega Biotech Iberica S.L.) for 10 min before centrifuging at 160 × g for 10 min. All procedures were conducted at room temperature (RT). Neutrophil viability was determined by trypan blue (Sigma-Aldrich) exclusion. Neutrophil purity and count were determined by extension and staining, by flow cytometry and by quantification with the automatic hematology analyzer Sysmex^®^ Serie-XN- BF.

### 2.6 Flow cytometry

Purity of isolated neutrophils was assessed using a combination of four monoclonal antibodies. An aliquot of 100 µL isolated neutrophils were stained with 5 µL of 6.3 μg/mL CD15-Pacific Blue, 10 µL of 50 μg/mL CD45-FITC, 10 µL of 12.5 μg/mL CD11b-PE and 5 µL of 12.5 μg/mL CD66b-APC (Beckman Coulter, Bre, CA, USA) for 15 min and then erythrocytes were lysed using FACS Lysing Solution (BD Biosciences, San Jose, CA). Cells were finally resuspended in 1X PBS and processed in the flow cytometer. At least 50000 events were acquired in a FACSCanto-II flow cytometer using the BD FACSDiva software (Becton Dickinson, San Jose, CA). Further analyses were performed with Infinicyt™ software (Cytognos, S.L., Salamanca, Spain).

### 2.7 NET-degradation assay

NET degradation was analyzed according to Jiménez-Alcázar et al. (24) with modifications. Purified neutrophils in Dulbecco’s Modified Eagle’s Medium (DMEM, Gibco, Life Technologies) were seeded to 96-well plates (Greiner Bio-One, Kremsmünster, Austria) at a concentration of 1 x 10^4^ cells/well. After a 1 h-incubation, neutrophils were activated with 100 nM phorbol 12-myristate 13-acetate (PMA; Sigma-Aldrich) for 4 h at 37°C with 5% CO_2_ and humidity. NETs were kept at 4°C overnight.

To test the stability of NETs in the presence of plasma, these were incubated with 5% citrated plasma samples from BC patients or healthy controls in HBSS++ (Gibco, Life Technologies) containing 20 µM thrombin inhibitor D-phenylalanyl-prolyl-arginyl chloromethyl ketone (PPACK; Santa Cruz Biotechnology, Heidelberg, Germany) for 6 h at 37°C with 5% CO_2_ and humidity. NET degradation was stopped by replacing the diluted plasma with 2% paraformaldehyde (PFA; Sigma-Aldrich) in 1X PBS. After incubation for 1 h at RT and washing twice with 1X PBS, DNA from nuclei and NETs were then labelled fluorescently by adding 5 nM Sytox Green (Invitrogen, Life Technologies) in 1X PBS. Total fluorescence of each well was quantified using a fluorometer (Fluoroskan, Thermo Fisher Scientific) with 482 nm excitation and 518 nm emission. Images of fluorescently stained nuclei and NETs were acquired with an inverted fluorescence microscope (Leica, Wetzlar, Germany).

### 2.8 *In vitro* therapeutic evaluation of DNaseI

To evaluate the potential therapeutic restoration of plasma DNaseI deficiency, *in vitro* formed NETs were incubated with 5% citrated plasma as previously described and supplemented with either DNaseI (TURBO DNA-freeTM Kit, Ambion, Life Technnologies) or the recombinant human DNaseI (rhDNaseI; Dornase alfa, Pulmozyme^®^; Roche, Mannheim, Germany), an approved drug for cystic fibrosis. Briefly, 5 µL DNaseI in 1X PBS containing 0.1% BSA (Sigma-Aldrich) to a final concentration of 500 µU/mL was added to each well. Supplementation with 1X PBS containing 0.1% BSA (vehicle) was used as negative control. After 6 h-incubation, NET degradation was stopped, nuclei and NETs were labelled, and DNA fluorescence was recorded as aforementioned.

### 2.9 Immunofluorescence staining of NETs in cell culture

The occurrence of NETosis was confirmed in PMA-activated neutrophils used for the NET degradation assay by immunostaining of NET-specific markers: NE and citrullinated histone 3 (citH3). A concentration of 1x10^5^ cells/well were seeded in 24-well plates containing cover slides. After a 1 h-incubation, neutrophils were activated with 100 nM PMA for 4 h at 37°C with 5% CO_2_ and humidity. NETs were kept at 4°C overnight.

Later, cells were fixed with 4% PFA in 1X PBS. After incubation for 30 min at RT and thoroughly washing with 1X PBS, 0.2% Triton X-100 (Sigma-Aldrich) in 1X PBS was added to permeabilize the cells for 5 min. After washing thoroughly with 1X Tris PBS, samples were blocked for 1 h at RT in 1X PBS supplemented with 10% normal goat serum (Sigma-Aldrich) and 0.1% Triton X-100. After thoroughly washing with 1X PBS, samples were incubated for 2 h at RT with mouse anti-NE (1:150; Sigma Aldrich, Missouri, USA) and rabbit anti-citH3 (1:50; Abcam) in 1X PBS supplemented with 10% normal goat serum. After thoroughly washing with 1X PBS, samples were incubated for 1 h at RT with a mix of secondary antibodies Alexa 488 goat anti-mouse (1:500) and Alexa 594 goat anti-rabbit (1:500) (Invitrogen, Life Technologies) in 1X PBS supplemented with 10% normal goat serum. After thoroughly washing with 1X PBS, samples were incubated for 10 min with Hoechst (1:1000 in 1X TBS, Invitrogen, Life Technologies) and finally Vectashield^®^ mounting media (Vector Laboratories. Burlingame, USA) was added. Images of fluorescently stained nuclei and NETs were acquired with an inverted fluorescence confocal microscope (Leica).

### 2.10 Immunofluorescence staining of NETs in tissue specimens

For each FFPE tissue specimen, the position of tumor and paratumor tissue was determined according to the results of hematoxylin and eosin staining. Five µm tissue sections were collected into Superfrost Plus Adhesion Microscope slides (Epredia, Braunschweig, Germany), incubated at 60°C for 25 min and then rehydrated by 5 min immersions in several solutions: twice Xylol, twice 100% ethanol, 96% ethanol, 70% ethanol, dH_2_O. Later, sections were soaked in sodium citrate buffer pH 6 at 90°C for 25 min and let cool, and then rinsed thoroughly with dH_2_O and 1X TBS. Then, 0.5% Triton X-100 in 1X TBS was added to permeabilize the tissue. Subsequent steps were performed as abovementioned with the following modifications: all steps were performed in 1X TBS; primary antibodies were incubated overnight at 4°C, being mouse anti-NE (1:50; Bio-Techne R&D, Minneapolis, USA); and after washing the secondary antibodies, samples were incubated for 1 h at RT with an autofluorescence quenching solution (CuSO_4_ 2 mM, NH_4_CH_3_CO_2_ 50 mM, pH 5.0).

NETs were counted with a 40X objective in 10 fields of view (221.7 × 165.2 µm). For quantification of NET formation, two distinct criteria had to be met in our experiments (1): presence of citH3 and (2) citH3 had to originate from cells staining positive for NE. Only if both criteria were fulfilled, the structure was defined as NET and included in the quantification.

### 2.11 Statistical analysis

The normality of the data was checked by a Shapiro-Wilk normality test. Continuous variables are presented as median and interquartile range (IQR). Categorical variables are presented as count and percentage. The results of experiments performed in replicates are reported as means ± standard error of the mean (SEM) unless otherwise stated. For unpaired comparisons of two groups, a t test and, in the case of non-normality, a non-parametric Mann-Whitney test was performed. For more than two unpaired groups, a variance analytical approach was used, and comparison was done with a one-way ANOVA, and Kruskal-Wallis test in case of non-normality. The correlation between NETs markers was assessed with a Spearman test. Graphpad Prism (v.9) was used to perform all analysis. Results were considered statistically significant at *P*<0.05.

## 3 Results

### 3.1 Clinical characteristics of the study subjects

The clinical characteristics of the study subjects are depicted in **Table 1**. As the incidence of BC is greater in males than in females, our cohort comprised a larger proportion of males in both clinical groups. BC patients had a higher neutrophil count than controls (*P*<0.001) but a similar neutrophil/lymphocyte ratio (*P*=0.238).

**Table 1:** Clinical characteristics of the BC patients and healthy controls studied.

	BC patients		Controls
	Plasma (n=71)	Tissue (n=30)	Plasma (n=64)
**Age, *y (IQR)***	68 (62-74)	67 (60-76)	64 (57-69)
**Male sex, *N (%)***	59 (83.1)	27 (90.0)	53 (82.8)
**Neutrophil count, *median (IQR)***	5.0 (3.7-6.7)	4.7 (3.7-6.3)	3.8 (3.0-4.5)
**Lymphocyte count, *median (IQR)***	1.9 (1.6-2.5)	2.3 (1.8-2.6)	1.8 (1.5-2.1)
**Neutrophil/lymphocyte ratio, *median (IQR)***	2.2 (1.7-3.6)	2.2 (1.6-2.8)	2.1 (1.5-2.8)
**Tumor Stage and Grade, *N (%)*** **TaG1** **TaG2** **TaG3** **T1G2** **T1G3** **pTis** **T2G2** **T2G3** **T3G2** **T3G3** **T4G2** **T4G3** **T4G4**	5 (7.1) 20 (28.2) 3 (4.2) 1 (1.4) 12 (16.9) 2 (2.8) 1 (1.4) 10 (14.1) 1 (1.4) 8 (11.3) 2 (2.8) 5 (7.0) 1 (1.4)	10 (33.3) 0 (0) 6 (20.0) 0 (0) 5 (16.7) 0 (0) 0 (0) 9 (30.0) 0 (0) 0 (0) 0 (0) 0 (0) 0 (0)	- - - - - - - - - - - - -
**Type of surgery, *N (%)*** **TURBT** **Partial cystectomy** **Radical cystectomy**	43 (60.6) 1 (1.4) 27 (38.0)	30 (100) 0 (0) 0 (0)	- - -
Disease status until the end of the follow-up*, *N (%)* **disease-free alive** **progression alive** **death by BC** **death by other**	51 (71.8) 9 (12.7) 11 (15.5) 0 (0)	17 (56.7) 4 (13.3) 5 (16.7) 4 (13.3)	- - - -
**Tumor origin, *N (%)*** **primary** **recurrent** **unknown**	31 (43.7) 27 (38.0) 13 (18.3)	29 (96.7) 1 (3.3) 0 (0)	- - -
**MIBC progression from NMIBC, *N (%)***	12 (16.9)	2 (6.7)	–
**Hematuria, *N (%)*** **at diagnosis** **at sampling**	59 (83.1) 4 (5.6)	26 (86.7) 21 (70)	- -
**Tumor size, cm^2^** **<1** **1-3** **>3**	3 (4.2) 41 (57.8) 27 (38.0)	0 (0) 19 (63.3) 11 (36.7)	- - -
**Tumor multifocality, *N (%)*** **1** **2-3** **>3**	41 (57.7) 22 (31.0) 8 (11.3)	22 (73.3) 6 (20) 2 (6.7)	- - -
**Previous treatment, *N (%)*** **doxorubicin** **mitomycin** **BCG** **combined treatments** **none**	1 (1.4) 2 (2.8) 6 (8.5) 5 (7.0) 57 (80.3)	0 (0) 0 (0) 1 (3.3) 0 (0) 29 (96.7)	- - - - -
**Tumor histology** **papillary** **concomitant CIS**	64 (90.1) 7 (9.9)	30 (100) 0 (0)	- -
**Smoking, *N (%)*** **no** **former** **current** **unknown**	12 (16.9) 15 (21.1) 41 (57.8) 3 (4.2)	3 (10) 13 (43.3) 14 (46.7) 0 (0)	- - - -

Continuous variables are presented as median and interquartile range (IQR). Categorical variables are presented as count and percentage. * Participants were clinically followed-up until May 2020. BC, bladder cancer; MIBC, muscle-invasive bladder cancer; NMIBC, non-muscle-invasive bladder cancer; TURBT, transurethral resection of bladder tumor; BCG, Bacillus Calmette-Guerin; CIS, carcinoma *in situ*.

### 3.2 Plasma NETs and inflammatory markers and DNaseI activity

**Figure 1:**
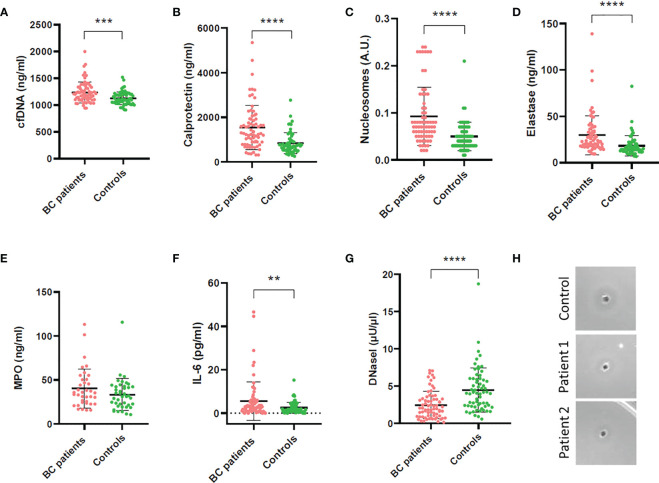
Quantification of NETs and inflammatory markers and DNaseI activity in plasma of BC patients and controls. **(A)** Cell-free DNA; **(B)** Calprotectin; **(C)** Nucleosomes; **(D)** Neutrophil elastase (NE); **(E)** Myeloperoxidase (MPO); **(F)** Interleukin 6 (IL-6); **(G)** DNaseI activity measured by the single radial enzyme-diffusion (SRED) assay; **(H)** Degradation halo formed by DNaseI activity in the single radial enzyme-diffusion (SRED) assay. **P<0.01*;* ***P<0.001; ****P<0.0001.

**Figure 2:**
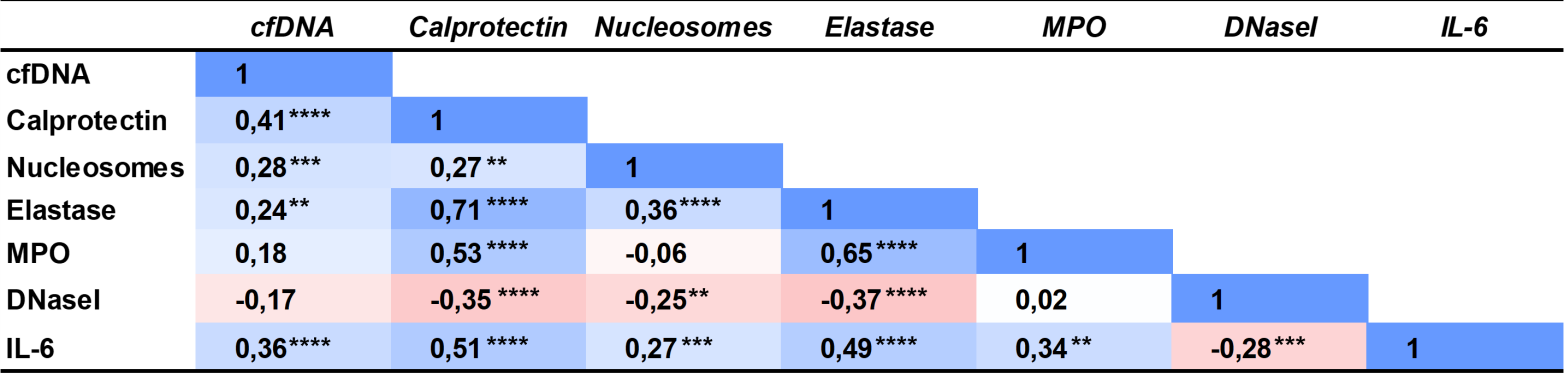
Spearman correlation coefficient matrix of the NETs markers, DNaseI activity and interleukin 6 measured in plasma of BC patients and controls. **P<0.01; ***P<0.001; ****P<0.0001.

BC patients had significantly increased NETs markers in plasma compared to controls (overall *P*<0.0001; **Figure 1**). Particularly, patients had increased cfDNA levels (median 1199 ng/mL, 1^st^-3^rd^ quartiles 1101-1333) compared to controls (1102 ng/mL, 1049-1207) *(P*=0.0003; **Figure 1A**); calprotectin: 1271 ng/mL (782.3-1921.0) *vs.* 720.3 ng/mL (533.63-957.3) *(P*<0.0001; **Figure 1B**); nucleosomes: 0.070 A.U. (0.050-0.110) *vs.* 0.040 A.U. (0.030-0.0675) (*P*<0.0001; **Figure 1C**); and NE: 22.72 ng/mL (17.96-34.17) *vs.* 15.18 (12.19-20.40) (*P*<0.0001; **Figure 1D**). We measured MPO in 37 BC patients and 41 controls but then the kit employed was discontinued. We ceased its measurement, as no substantial differences arose between patients (35.49 ng/mL; 24.14-50.53) and controls (31.22 ng/mL; 20.93-42.57) (*P*=0.1643; **Figure 1E**). A significant correlation was found among all NETs markers studied, suggesting that neutrophils may be the origin of these markers in plasma (**Figure 2**).

The inflammatory marker IL-6 was also increased in BC patients (2.650 pg/mL; 1.020-5.910) compared to controls (1.610; 0.8400-3.773) (*P*=0.0188; **Figure 1F**) and it correlated with all NETs markers in plasma (**Figure 2**).

We measured DNaseI activity in plasma of BC patients and controls, to evaluate whether the increased NETs evidenced were due to an impaired NET degradation. Remarkably, the DNaseI activity was significantly lower in BC patients (1.97 μU/μL; 0.93-3.28) than in controls (4.05; 2.32-6.00) (*P*<0.0001; **Figure 1G**), as seen in the activity halo (**Figure 1H**). Interestingly, all NETs markers inversely correlated with DNaseI activity but cfDNA (**Figure 2**), as expected, since the low DNaseI observed may be insufficient to degrade all the NET cfDNA released.

### 3.3 Immunofluorescence staining of NETs in BC tissue

**Figure 3:**
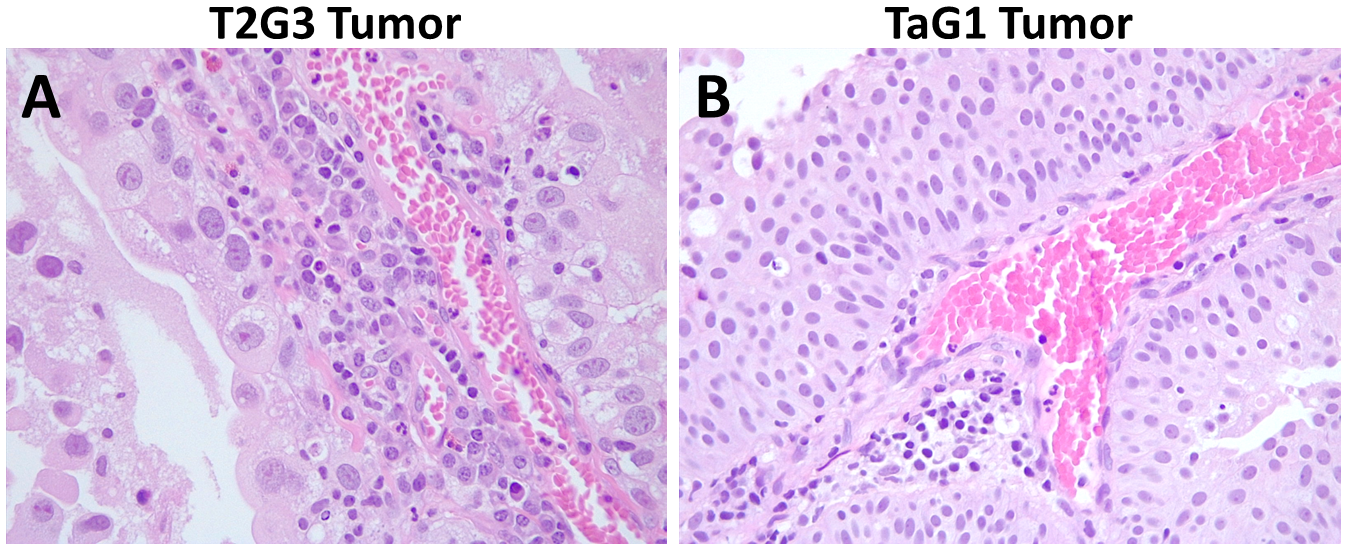
Hematoxylin and eosin staining of formalin fixed and paraffin embedded BC carcinomas. **(A)** Representative image of a T2G3 tumor. **(B)** Representative image of a TaG1 tumor. Pictures were taken with a 40X objective.

We evidenced the presence of a high neutrophil infiltration in MIBC FFPE tissue specimens compared to NMIBC. As seen in **Figure 3A**, the T2G3 papillary urothelial carcinoma reveals a dense inflammatory infiltrate in which numerous extravasated neutrophils and mononuclear cells are probably interacting with tumor cells, whereas the TaG1 tumor (**Figure 3B**) displays a mild inflammatory response within the tumor papillae with scanty mononuclear and neutrophilic cells in close relationship with tumor cells.

**Figure 4:**
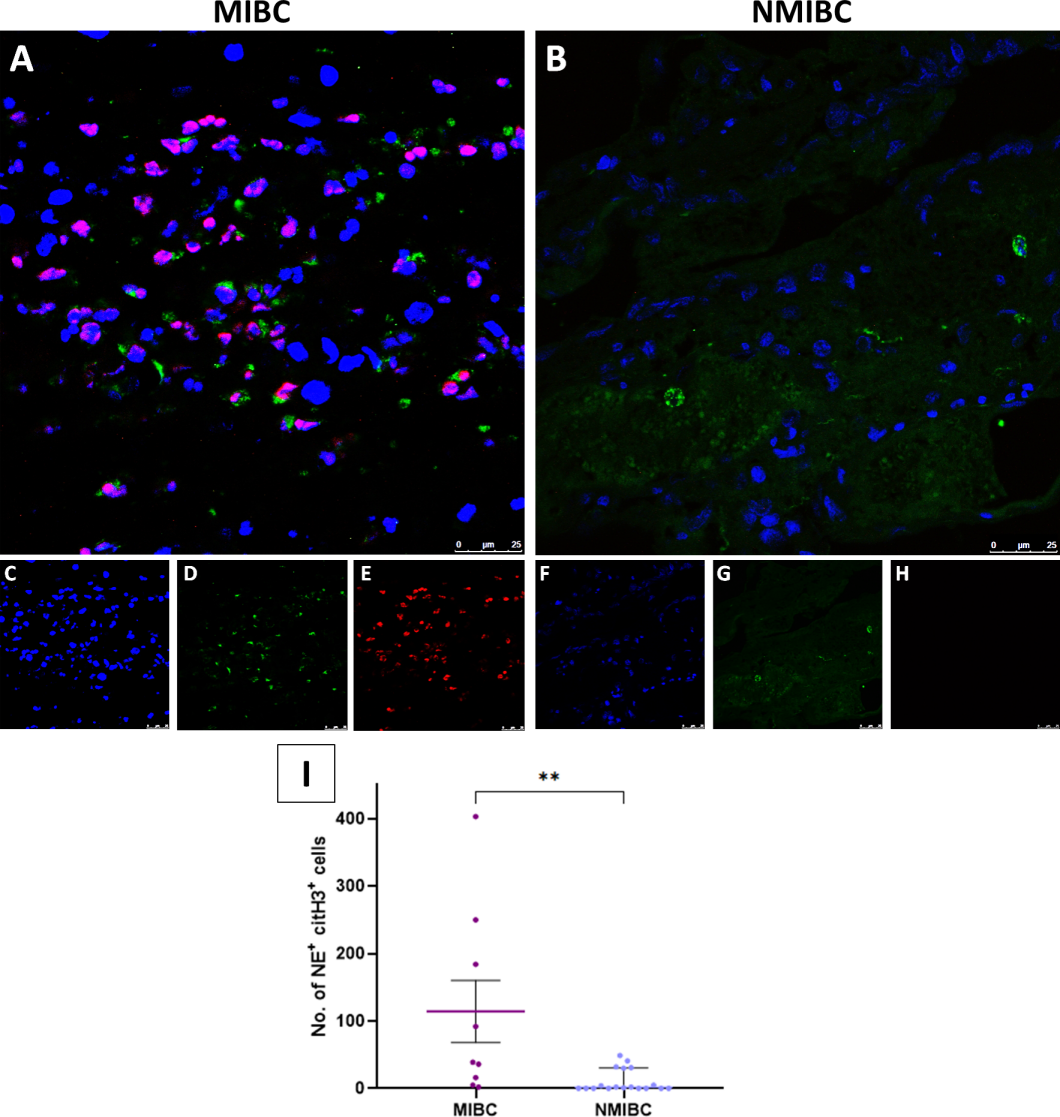
Immunofluorescence staining of NETs in FFPE BC tissue sections. **(A)** Colocalization of neutrophil elastase (NE) and citrullinated histone H3 (citH3) with the DNA extracellular fibers in the tumor tissue from a muscle-invasive bladder cancer (MIBC) patient; **(B)** Colocalization of NE, citH3 and DNA in the tumor tissue from a non-muscle invasive bladder cancer (NMIBC) patient; **(C)** DNA (Hoechst); **(D)** NE; **(E)** citH3; **(F)** DNA; **(G)** NE; **(H)** citH3; **(I)** Number of NET formations in 10 fields in the tumor tissue samples. Pictures were taken with a confocal microscope 40X objective. **P<0.01.

To prove the occurrence of NETosis, we performed immunofluorescence staining of FFPE specimens from 30 BC patients of different grades and stages (**Table 1**). We confirmed the existence of NETosis through specific co-localization of citH3 and NE in the extracellular DNA fibers (**Figure 4**). Furthermore, we evidenced a significant increase in NETosis in MIBC patients (11; 0.5-59.5) compared to NMIBC patients (0, 0–1) (*P=*0.0051) (**Figure 4A** *vs.* **4B** and **4I**). No differences arose among NMIBC TaG1, TaG3 or T1G3 patients (*P*=0.8661; data not shown).

### 3.4 NETs degradation assay and *in vitro* therapeutic evaluation of DNaseI

**Figure 5:**
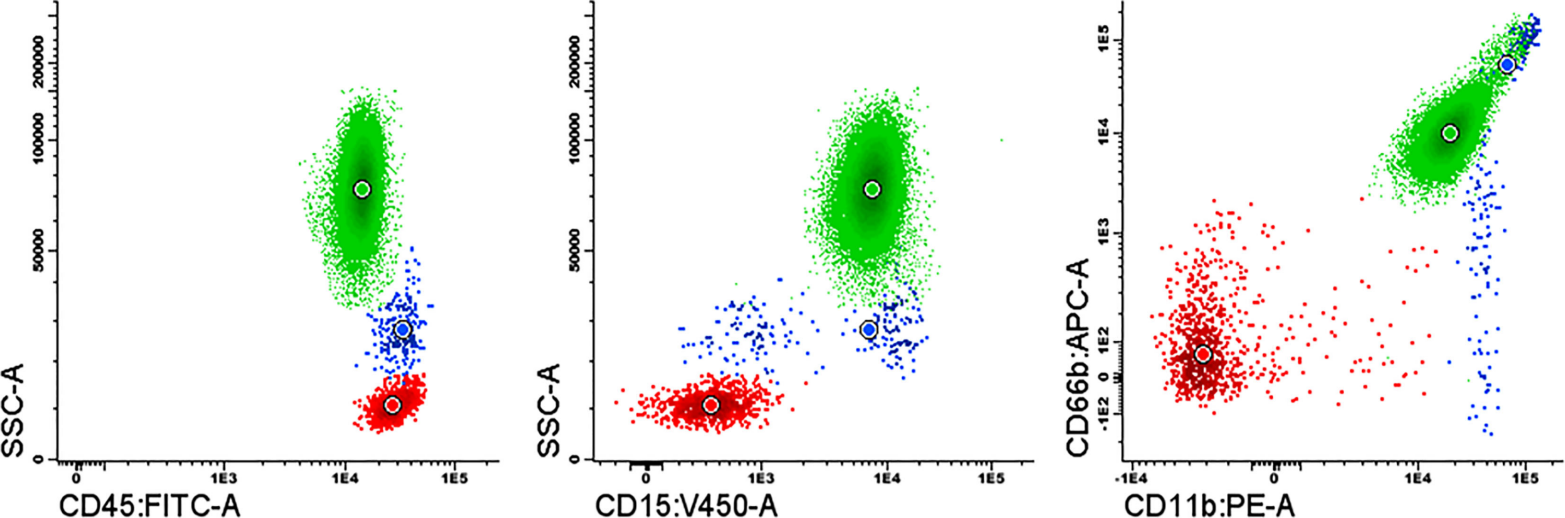
Purity assessment of the isolated neutrophils by flow cytometry using a combination of the monoclonal antibodies fluorochrome conjugated: CD15-Pacific Blue, CD45-FITC, CD11b-PE and CD66b-APC. Neutrophils (green); monocytes (blue); lymphocytes (red).

The purity of the neutrophils isolated from a healthy individual to form NETs was >97% assessed by flow cytometry (**Figure 5**).

**Figure 6:**
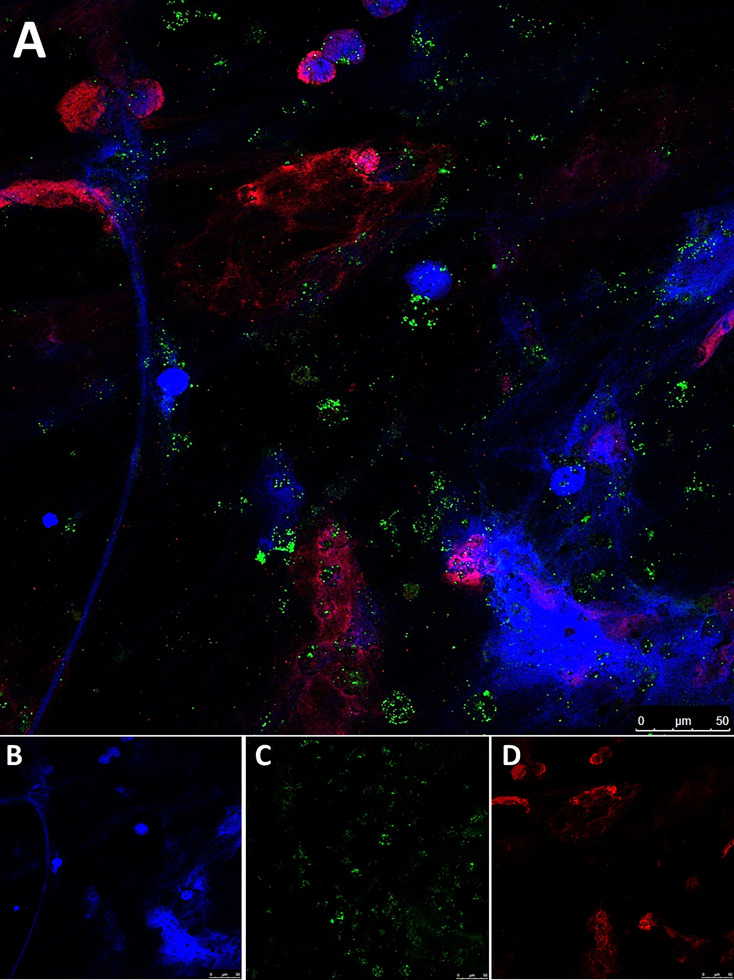
Immunofluorescence staining of NETs generated *in vitro*. **(A)** Colocalization of neutrophil elastase (NE) and citrullinated histone H3 (citH3) with extracellular DNA fibers in PMA-induced NETs from a healthy donor; **(B)** DNA (Hoechst); **(C)** NE; **(D)** citH3; Pictures were taken with a confocal microscope 40X objective.

After PMA-activation, we verified the occurrence of neutrophil activation through NETosis by immunofluorescence through specific co-localization of citH3 and NE with extracellular DNA fibers (**Figure 6**).

**Figure 7:**
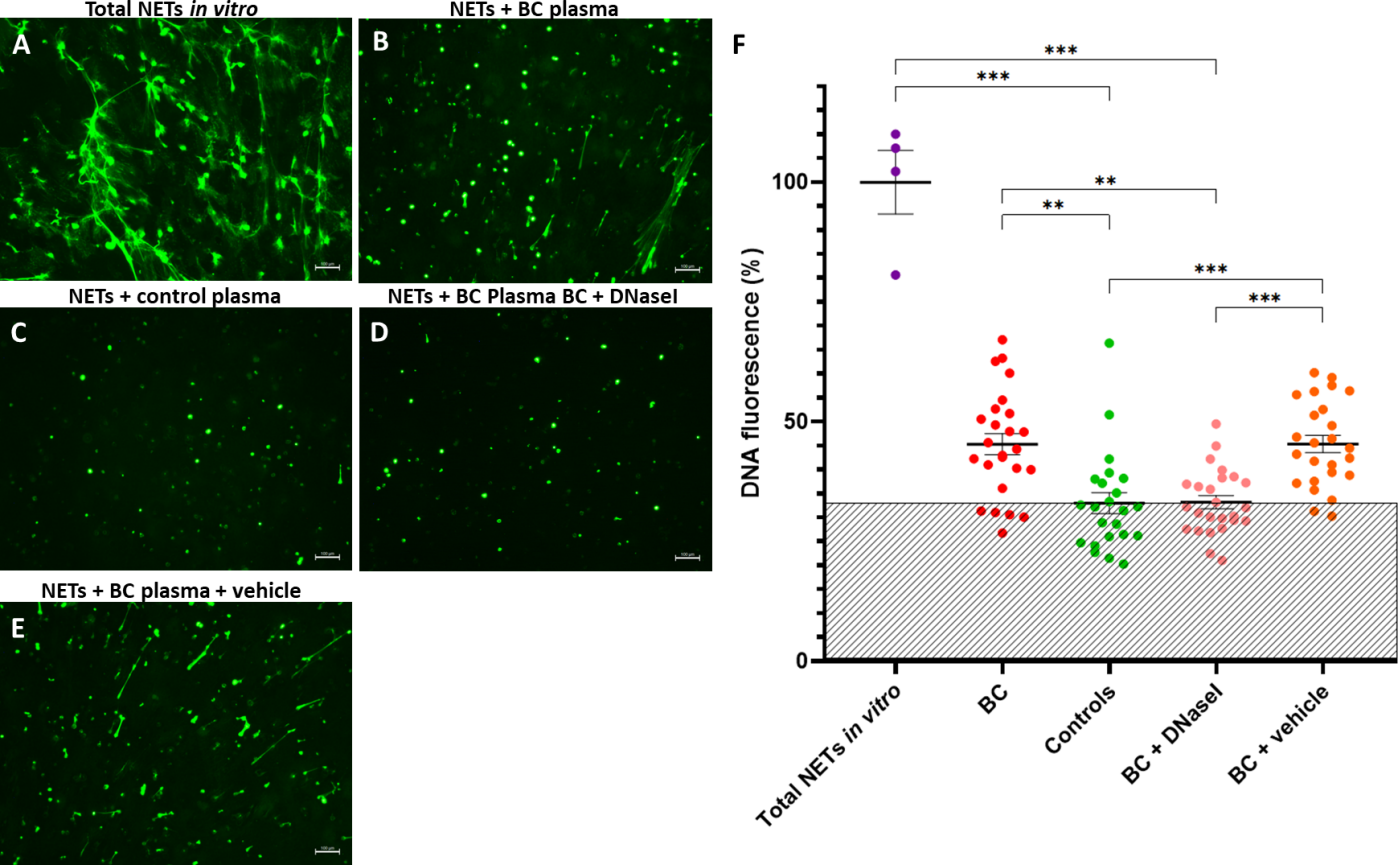
NET degradation experiments *in vitro*. **(A)** NETs generated *in vitro*; Remaining NETs after incubation with: **(B)** Plasma from a BC patient; **(C)** Plasma from a healthy donor (control); **(D)** Plasma from a BC patient supplemented with rhDNaseI (Dornase alfa, Pulmozyme^®^, Roche) in 1X PBS + 0.1% BSA; **(E)** Plasma from a BC patient supplemented with vehicle (1X PBS + 0.1% BSA). The green fluorescence was obtained by addition of Sytox Green and pictures were taken with an inverted fluorescence microscope 10X objective; **(F)** DNA fluorescence after NET degradation experiments *in vitro*. Shaded area depicts the mean DNA fluoresce of controls. **P<0.01, ***P<0.001.

Isolated neutrophils were forced to form NETs *in vitro* (**Figure 7A**) and incubated with plasma from a subset of 25 BC (9 NMIBC and 16 MIBC patients) and 23 controls to ascertain the ability of plasma to degrade NETs, assessed by fluorescence microscopy and by measuring the DNA fluorescence. Incubation with plasma did eliminate elongated NETs (extracellular DNA fibers) but not nuclei as dots. Interestingly, plasma of BC patients had an impaired ability to degrade NETs compared to that of controls, since NETs were still visible (**Figures 7B, C**). Indeed, compared to NETs incubated with buffer, the DNA fluorescence after the incubation with BC plasma only decreased to 45.3%, while it decreased to 33% when incubated with plasma from healthy controls (*P*<0.001) (**Figure 7F**).

Next, we evaluated the potential therapeutic restoration of the DNaseI deficiency observed in BC plasma with the rhDNaseI Dornase alfa as potential treatment. Remarkably, the addition of rhDNaseI Dornase alfa to BC plasma restored its NET degradation ability up to the level of healthy controls (**Figures 7D, C**). Thus, compared to NETs incubated with buffer, the DNA fluorescence after the supplementation of BC plasma with Dornase alfa was lower (33.2%) than that obtained without supplementation (45.3%) (*P*<0.001) and similar to that of control plasma (33%) (*P*>0.9999) (**Figure 7F**). Similar results were obtained when synthetic DNaseI was added (data not shown). Furthermore, the supplementation of BC plasma with vehicle did not affect its NET degradation ability (**Figures 7B, E**) (45.3% *vs*. 45.4%).

**Figure 8:**
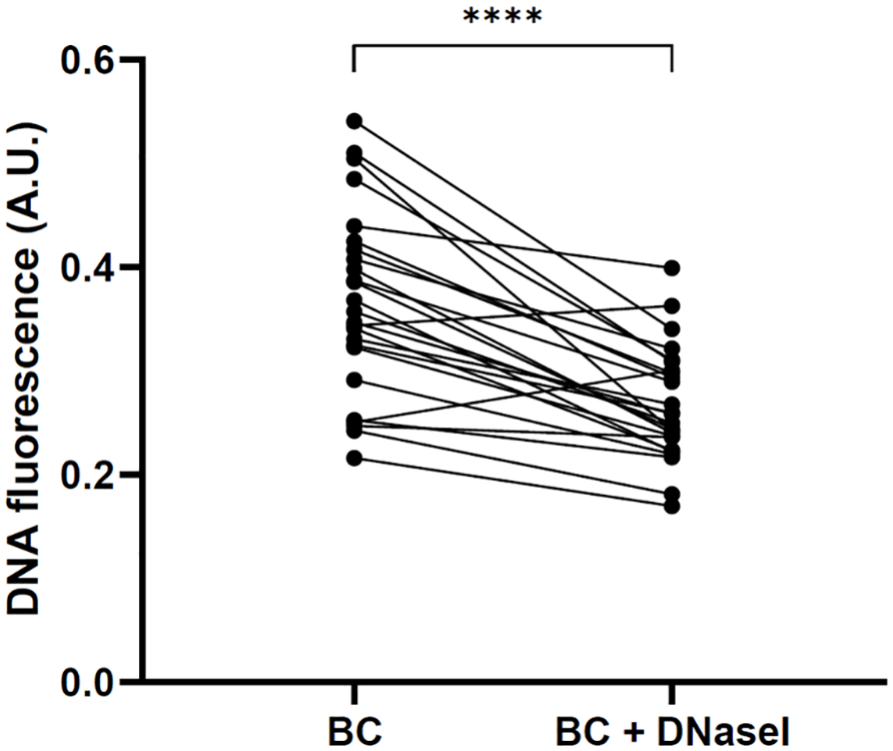
Restoration of NET degradation ability of BC plasma after the supplementation with rhDNaseI (Dornase alfa, Pulmozyme^®^, Roche). Changes in DNA fluorescence in NET degradation experiments *in vitro* after the incubation with plasma from each BC patient and with plasma from the same patients supplemented with rhDNaseI. ****P<0.0001.

Taking a close look at the behavior of each patient, we could observe that all patients restored the NET degradation ability with the addition of rhDNaseI, suggesting that the NET degradation impairment of BC patients is not caused by DNaseI inhibitors or anti-NET antibodies (**Figure 8**).

## 4 Discussion

The role of neutrophils in tumor development is well recognized as the neutrophil count is increasingly being considered as a marker of tumor progression and patient outcome. However, there is increasing evidence showing that neutrophils play a direct role in tumor growth beyond being a mere biomarker of worse prognosis (25). Cancer induces the ideal environment for NET formation, also promoting a prothrombotic state (10–12, 26–28), but the occurrence of NETosis in BC patients has never been ascertained.

We have evidenced a significant increase in blood neutrophil count and NETs markers in plasma of BC patients compared to that of healthy controls, namely cfDNA, calprotectin, nucleosomes and NE. Although cfDNA may also derive from cell death other than NETosis (29) a significant correlation was found between all NETs markers studied, thus suggesting a common origin. Moreover, we have also observed a high neutrophil infiltration in BC tissue and evidenced the occurrence of NETosis in the BC tumor microenvironment, which is remarkably increased in MIBC patients, those with the worst prognosis. Our findings confirm the implication of NETs in BC at systemic and local level. Similarly, previous studies have evidenced an increase in NETs markers in plasma or serum of patients with cancer (11, 12, 30–33) venous thrombosis (13), thrombotic microangiopathies (24), abdominal aortic aneurysm (34), diabetes (35), lupus (36), or acute arthritis (37), among others, thus revealing the importance NETs may have in diagnosis, prognosis and patient management. Whether NETs markers would be suitable biomarkers to improve BC diagnosis or to predict the outcome of BC patients during follow-up should be addressed in future studies properly designed for that aim.

Increased levels of IL-6 have been evidenced in patients with several tumor types, highlighting the link between inflammation and cancer. In fact, IL-6 is one of the most important cytokines during tumorigenesis and metastasis, as it provides the tumor cells with the ability to escape cell death thus promoting tumor cell survival (38). Herein, we have evidenced an increase in plasma IL-6 levels in BC patients that correlate with all NETs makers studied. These results highlight the inflammatory state occurring, which can also exacerbate NET formation.

The structural backbone of the NETs scaffold is DNA, selectively degraded by plasma DNaseI (24, 36). Indeed, to effectively lyse a clot both tPA and plasma DNaseI are required to lyse the fibrin and DNA structures, respectively (8). Previous findings have revealed that DNaseI deficiency is responsible for several disorders. Hakkim et al. (36) demonstrated that lupus nephritis is caused by a prolonged NET half-life due to an impaired NET degradation, which is mediated by an impairment of DNaseI activity or to the presence of anti-NET antibodies that protect them from degradation by plasma DNaseI. In line, Jiménez-Alcázar et al. (24), showed that patients with thrombotic microangiopathies have increased NETs in plasma caused by an impaired plasma DNaseI activity, which may cause the persistence of pro-thrombotic NETs and thus promote microvascular thrombosis in these patients. Accordingly, we measured plasma DNaseI activity and found a significant decrease in BC patients compared to controls. This decreased DNaseI activity might be due to the consumption during excessive tumor-induced NETosis and cell death, and may be in part responsible for the increase in NETs found in plasma of these patients. In addition, mutations in, the gene encoding for DNaseI (*DNASE1*) could also cause a reduction in the levels of plasma DNaseI in a subset of BC patients, as occurs for some patients with systemic lupus erythematosus who develop lupus nephritis (39), a fact that should be addressed in future studies. To further prove this effect, we isolated neutrophils from a healthy donor, prompted NETosis and verified its occurrence by immunofluorescence co-staining of citH3, NE and DNA. Then, we incubated NETs with plasma from BC patients and controls, and we validated that BC plasma has an overall 55% impaired ability to degrade NETs. Recently, Oklu et al. (40) evidenced an increase in plasma cfDNA associated with a decreased DNaseI activity in cancer patients, which reinforces our findings. The supplementation of BC plasma with rhDNaseI restored the ability to degrade NETs, which indicates that impaired NET degradation in BC patients is not due to DNaseI inhibitors or anti-NET antibodies as in some systemic lupus erythematosus patients (36), but a consequence of reduced levels of plasma DNaseI. Whether a small subset of BC patients have antibodies against NETs components such as MPO (41) or DNaseI inhibitors in plasma, should be explored in future studies.

Subsequently, we aimed to move one step forward and we tried to explore *in vitro* the therapeutic restoration of the DNaseI deficiency occurring in BC patients’ plasma. For that aim we employed the rhDNaseI Dornase alfa (Pulmozyme^®^, Roche), a drug currently employed inhaled for the treatment of cystic fibrosis as it is able to reduce the viscoelasticity of the sputum by breaking the long cfDNA molecules, thus improving the patient´s clinical response (42). We repeated the NET degradation assay but supplementing the patient´s plasma with Pulmozyme^®^ and we verified that we could restore the NET degradation ability up to the level of healthy controls. Accordingly, the approved drug Pulmozyme^®^ may represent a novel therapeutic tool for BC. This evidence opens a new venue for the local treatment of BC through the instillation of rhDNaseI into the bladder to compensate for the DNaseI reduction, limit the NETosis in the tumor microenvironment and reduce the possibility of tumor progression and metastasis while reducing the risk of cancer-associated thrombosis in these patients. Targeting NETs as therapeutic objective is on focus (43), also for BC (22, 23). Recently, Alekseeva et al. (44) demonstrated in a murine model of metastatic B16 melanoma that Pulmozyme^®^ administered intranasally or intramuscularly strongly reduced tumor cell migration and induced apoptosis of B16 cells *in vitro* and effectively inhibited metastases in lungs and liver *in vivo*. Particularly, Pulmozyme^®^ enhanced the blood DNase activity of melanoma mice to the level of healthy animals, significantly decreased serum cfDNA concentrations, efficiently degraded DNA fragments in the bloodstream and induced apoptosis and disintegration of NETs in metastatic foci; as a result, this manifested as the inhibition of metastases spread. These authors suggest the use of Pulmozyme^®^ to treat lung metastases. Presently, Pulmozyme^®^ is being tested in clinical trials for the treatment of ischemic stroke (NCT05203224, NCT04785066), COVID-19 (NCT04541979, NCT04409925), and dry eyes (NCT02193490), among others; but yet none focus on cancer (https://clinicaltrials.gov/ct2/results?cond=&term=Pulmozyme&cntry=&state=&city=&dist=).

A limitation of our study is the absence of healthy bladder urothelium specimens analyzed. However, the tissue surrounding a bladder tumor cannot be considered healthy as it contains genetic alterations, and bladder urothelium from healthy individuals is almost impossible to obtain. Strengths of our study are a thorough evaluation of patients at inclusion and during follow-up, the study of all stages of BC patients and the inclusion of healthy volunteers with clinical evidence of absence of urological disorders. Our positive results endorse the performance of validation studies in animal models of BC.

In conclusion, neutrophils release NETs which enhance tumor growth and metastasis. We have firstly demonstrated the occurrence of an increased NETosis in BC patients both systemically and in the tumor microenvironment, which is partly caused by a reduction in plasma DNaseI. More importantly, we have demonstrated that this deficiency can be restored with rhDNaseI to the level of healthy controls, opening new venues for BC treatment. All in all, the potential use of Pulmozyme^®^ as therapeutic tool to reduce NETosis in high-risk MIBC patients may ameliorate cancer progression, metastasis and cancer-associated thrombosis improving patient outcome.

## Data availability statement

The raw data supporting the conclusions of this article will be made available by the authors, without undue reservation.

## Ethics statement

The studies involving human participants were reviewed and approved by the ethics review board at la Fe University and Polytechnic Hospital (ref. 2014/0314). The patients/participants provided their written informed consent to participate in this study.

## Author contributions

Study concept and design: RH, JO and PM. Acquisition of data: RH, JO, MH, FC, MC, LC, MM-S and CV-D. Analysis and interpretation of data: RH, JO, MH, LC and PM. Drafting of the manuscript: RH and PM. Critical revision of the manuscript for important intellectual content: JO, EP, SB, CV-D and MM-S. Statistical analysis: RH. Obtaining funding: EP and PM. Administrative, technical or material support: FC and DR-S. Supervision: PM. All authors have read and approved the final version of the manuscript.
